# How does the community COVID-19 level of risk impact on that of a care home?

**DOI:** 10.1371/journal.pone.0260051

**Published:** 2021-12-31

**Authors:** Glenna Nightingale, Megan Laxton, Janine B. Illian

**Affiliations:** 1 School of Health in Social Sciences, University of Edinburgh, Edinburgh, United Kingdom; 2 School of Mathematics and Statistics, University of Glasgow, Glasgow, United Kingdom; University Magna Graecia of Catanzaro, ITALY

## Abstract

**Objectives:**

To model the risk of COVID-19 mortality in British care homes conditional on the community level risk.

**Methods:**

A two stage modeling process (“doubly latent”) which includes a Besag York Mollie model (BYM) and a Log Gaussian Cox Process. The BYM is adopted so as to estimate the community level risks. These are incorporated in the Log Gaussian Cox Process to estimate the impact of these risks on that in care homes.

**Results:**

For an increase in the risk at the community level, the number of COVID-19 related deaths in the associated care home would be increased by exp (0.833), 2. This is based on a simulated dataset. In the context of COVID-19 related deaths, this study has illustrated the estimation of the risk to care homes in the presence of background community risk. This approach will be useful in facilitating the identification of the most vulnerable care homes and in predicting risk to new care homes.

**Conclusions:**

The modeling of two latent processes have been shown to be successfully facilitated by the use of the BYM and Log Gaussian Cox Process Models. Community COVID-19 risks impact on that of the care homes embedded in these communities.

## Introduction

The reported numbers of COVID-19 related deaths in care homes worldwide have been a startling indication of the vulnerability of care homes to disease outbreaks [1–3] and has highlighted the need for the statistical policy instruments which can allow predictions with an associated measure of uncertainty. Internationally, the impact of this disease on care homes has been reported to be severe. In particular, the total “share” of COVID-19 related deaths in care homes was reported to be highly correlated to COVID-19 deaths in the community within which the care homes are located [4]. One issue which has been highlighted in relation to making comparisons across countries, is that there exist differences in the definitions of care homes, and data collection on COVID-19 mortality varies across countries.

In the UK, studies based on data from Wales, and separately from Scotland point to a statistically significant association between COVID-19 deaths in care homes and size of care home [5, 6]. The study based on Scottish data identified and reported on the transfer (or discharge) of patients from NHS hospitals to Scottish care homes. This work was commissioned by the Cabinet Secretary for Health. The study from Wales reveals that there is no evidence of a statistically significant association between the transfer of patients from hospitals to care homes. The Scottish study suggests that there is the presence of an association between the transfer of patients from hospitals to care homes. The study does indicate that the risk of an outbreak in a Scottish care home is greater when considering care home size (vs. transfer from hospitals). Others [7] report in a UK wide cohort-study that many people in care homes died from COVID-19 without being tested. Additionally, it was found that the risk of infection in care homes was impacted by low staff and separately, high occupancy.

The impact of the community on the observations at the care home level is also an important factor [2] and this is why we have adopted a two-stage modeling approach to facilitate the generation of relative risk estimates at the community level.

The proposed novel modeling approach is an important tool for estimating and predicting deaths related to COVID-19 at care homes in the presence of community level relative risk estimates. Methods commonly used include logistic regression models [7] or rate calculations which do not incorporate the spatial correlation between sites of observations such as a network of care homes.

The proposed method, “doubly-latent” modeling incorporates the fact that there are two latent processes at play: risk of COVID-19-related deaths at the community level, and also at the care home (or other spatial community) level. This approach is illustrated using simulated care home data, because of the paucity of suitable data. This approach can be applied to estimating disease risk in other spatial settings such as schools, nurseries, offices and gyms.

## Methods

The approach adopted here brings together the Besag York Mollie (BYM) model [8] and Log Gaussian Cox Processes [9] to estimate the impact of community disease risk on the risk to individual care homes within a given care home network.

The modeling approach is a two-stage process.

### Stage 1: Estimating community risk per administrative geographic region

In the first stage, the reported counts of COVID-19 related deaths by geographic region are used. This type of spatial data is classified as areal data because of its aggregated nature. A Besag York Mollie model [10] is then constructed so as to generate relative risk estimates associated with each geographic level, given selected covariates such as population size, and deprivation level. If this is done in a Bayesian framework, the estimates are really “posterior” mean estimates. These estimates can then be visualized through choropleth maps showing the relative risk per geographic region.

In the BYM framework, the number of COVID-19 related deaths per geographic area is the dependent variable *y*_1*i*_ with

$$y_{1i}∼Poisson(\eta_{i})$$

and the linear predictor in this case is:

$$\eta_{i}=\log(p_{i})=\alpha_{1}+\sum_{k=1}^{n_{\beta }}\beta_{1k}z_{ki}+\phi_{i}+\theta_{i}.$$

Here *α*_1_ is the intercept, the *β*_*k*_ are parameters relating the k = 1,…, *n*_*β*_ covariates to the response. The components *ϕ*_*i*_ and *θ*_*i*_ are random effect terms for structured spatial effects and independent and unstructured (i.e. independently and identically distributed) random effects, respectively. Here, the structured effect a spatial random effect follows a CAR distribution, where the value in one cell is dependent on its spatial neighbours [11] and the unstructured effect, a Gaussian distribution with mean 0 and variance, $\sigma_{\theta }^{2}$. The mean estimated counts of covid-19 deaths is then used to calculate the relative risk in each of the areas via the linear predictor.

The analyses are conducted using the R package R-INLA [12, 13]

### Stage 2: Estimating risk to individual care homes

In this stage, the spatial distribution of the observed counts of COVID-19 related deaths within the study timeframe, per care home is modeled along with the spatial location of the care homes as these are likely to not be randomly distributed in space.

Each care home is associated with one geographic area. The relative risk associated with this region is considered to be a covariate in the analysis and has been estimated through the BYM model, as described above.

Each care home is also associated with a count of COVID-19 deaths within the study period. The second modeling stage will use the spatial distribution of the observed COVID-19 related deaths per care home as the dependent variable while jointly modelling the spatial distribution of the care homes using a model with two likelihoods which share some components, specifically the spatially structured random field.

Technically, the set of the locations of the care homes (in addition to the COVID-19 related deaths per care home) can be interpreted as a marked point pattern, where the point pattern consists of the locations of the care homes and reflects their distribution in space and the “marks” are the counts of COVID-19 death associated with each care home.

### Log Gaussian Cox Processes

A marked Log Gaussian Cox Process (LGCP) will be used in the second modeling stage to model this marked point pattern. Log Gaussian Cox Processes [14, 15] are doubly stochastic models with two levels of stochasticity: a Poisson point process where the location of the points are independent, given a Gaussian random field that accounts for the underlying spatial correlation in the data. Common applications of LGCP models are modeling the occurrence of ecological organisms (or nests/home bases) in the presence of underlying environmental covariates such as vegetation or elevation, and spatial epidemiology [16, 17].

Overall, the model has the following form for the marks:

$$y_{2}(s)∼Poisson(\mu (s))$$

such that

$$\log(\mu (s))=\alpha_{2}+\beta_{12}(risk(s))+k\;\psi (s)+\omega (s)$$

and *y*_2_, α_2_, *β*_12_ represent the count of COVID-19 related deaths observed at a care home at location *s* (during the study period), the model intercept, and the model parameter which quantifies the impact of the relative risk in the geographic area in which the care home is located. The term *ψ* is a spatially structured random field that also appears in the linear predictor for the spatial pattern, *ω* denotes a spatially structured random field that is unique to the counts of COVID-19 deaths per care home, i.e. the “marks” of the point pattern), and *k* is a scaling factor.

The intensity of the point pattern is modelled as

$$\lambda (s)=\exp(\alpha_{3}+\psi (s)).$$

Here *α*_3_ is another intercept, and *ψ* is the same spatially structured random field as for the marks above; here the scaling factor is equal to 1 for identifiability reasons.

Both in stage 1 and in stage 2 the models are fitted using the computationally efficient fitting algorithm INLA (integrated nested Laplace approximation) and the random fields are approximated in continuous space using the SPDE approach introduced in [18]. The combination of the use of INLA with the SPDE approach allows us to fit the complex and computationally demanding models within a realistic time frame.

## Results and discussion

### Data visualization

**Fig 1:**
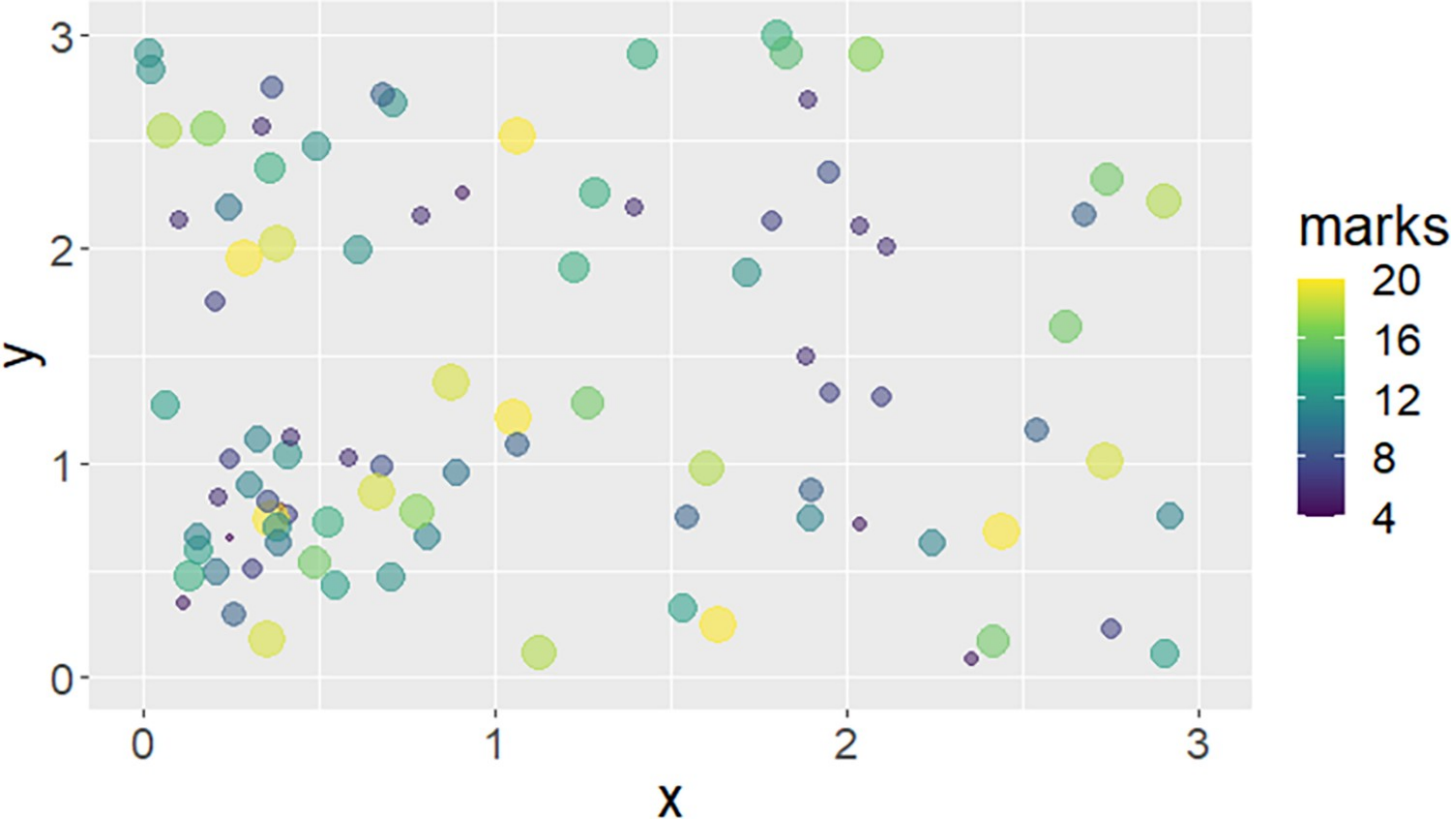
Marked point pattern of location of care homes. The size of each disc represents the relative count of COVID-19-related deaths associated with each care home. Each care home is associated with a mark (count of COVID-19-related deaths).

**Fig 2:**
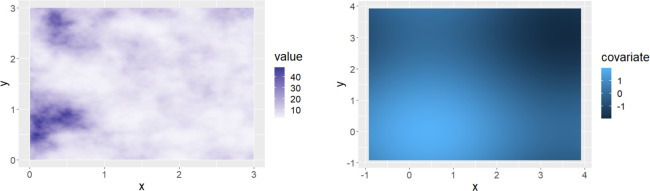
(a) The random field associated with the observed point pattern, (b) The map of the covariate: Relative risk estimates per geographic area.

The data for this study has been simulated using statistical software. The locations of simulated care homes are shown in Fig 1. These data can be classified as a marked point pattern where the “marks” in this case are the counts of COVID-19 related deaths. Fig 2 shows the location of simulated care homes by discs. The size (radius) and colour of each disc indicates the relative count of COVID-19 deaths observed at that specific care home.

The underlying random field which accompanies the location of care homes, is another data component in this study. This component is illustrated in Fig 2A. Guidance in simulating random field was obtained from Moraga et. al (reference book and Chapter 9). The random field in this study context would capture unobserved spatial correlation within the observed care home network.

Fig 2B illustrates the map of the covariate in this study. This has also been simulated and represents the continuous distribution of the relative risk estimates of COVID-19 related deaths in the community. This map would have been created using the relative risk estimates in stage 1 of the modeling process.

**Fig 3:**
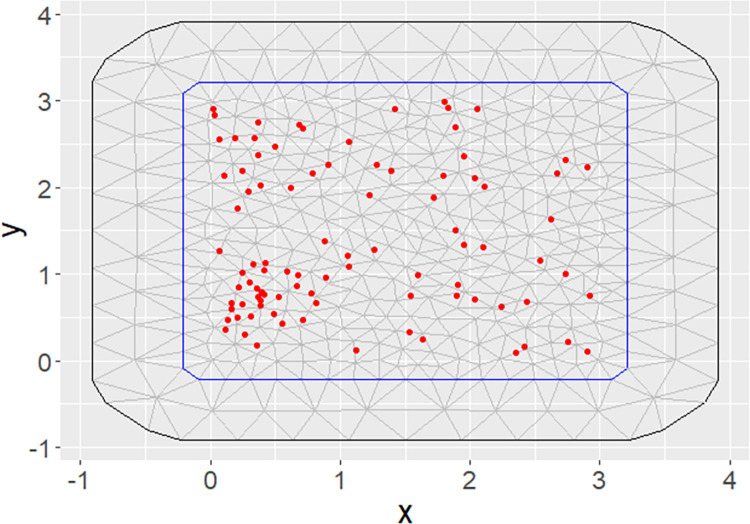
The interpolation grid for construction of the SPDE model.

Finally, Fig 3 shows the interpolation grid (or “mesh”) over which the SPDE model is constructed.

### Model estimates

Out of the two models considered, the model (Model 2) which contained both the community risk covariate and the spatial random field had a lower marginal log likelihood. This model estimates the effect of community level COVID-19 related risk of death on that for an individual care home in the said community. The random field estimates the effect of latent spatial variation on the risk to care homes. A summary of the model estimates is shown in Table 1. Recall that these results are based on data simulated for illustration of the method and are not based on actual data. In the model output we have parameterized the spatial fields for ease of discussion such that the range and standard deviation for the point field are denoted by ℵ_*p*_ and ℶ_*p*_ respectively, whilst that for the mark field, ℵ_*m*_ and ℶ_*m*_.

**Table 1:** Posterior means and 95% credible estimates for parameters (2.5% quantiles are #provided).

Parameters	Statistics	Model1	Model2
*β*_0_	mean	2.49	2.337
	2.5%	2.32	2.219
	97.5%	2.658	2.448
*β*_1_	mean		0.833
	2.5%		0.56
	97.5%		1.131
ℵ_*p*_		0.292	0.455
ℶ_*p*_		0.365	0.171
ℵ_*m*_		1.384	0.219
ℶ_*m*_		0.499	0.065

For the coefficient *β*_1_ in Model 2, the interpretation would be that for an increase in the risk at the community level, the number of COVID-19 related deaths in the associated care home would be increased by exp (0.833),2. This observation could be attributed to the fact that staff living in the community (and neighbouring communities) may be exposed to infection.

For the parameters relating to the spatial fields, the spatial range can be interpreted as the spatial distance over which counts of COVID-19 deaths per care home are correlated (or “connected”). The standard deviation of the spatial field gives an indication of variability associated with the spatial range. These two parameters are useful in the public health context because they provided the spatial context to disease spread.

If we consider Model 2, the estimate of the spatial range of the point field, exp(0.455) we can say that the distance across which the points are spatially correlated/connected is 1.58 spatial units. In terms of the mark field the distance across which the marks (counts of care home deaths) are spatially correlated/connected is 1.24 units.

**Fig 4:**
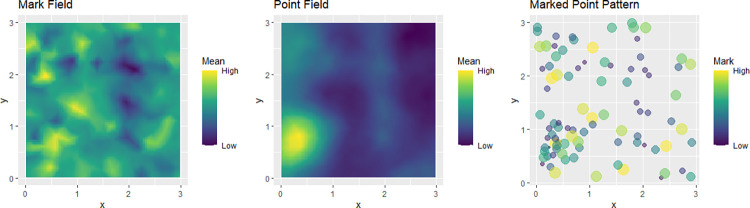
Multiplot showing the realization of the spatial fields alongside the marked point pattern.

Fig 4 is a multiplot which shows the realization of the spatial fields alongside the marked point pattern. In the figure we note that here is a higher degree of spatial clustering in the mark field. The spatial fields in this plot are generated from the model estimates.

In the context of COVID-19 related deaths, this study has illustrated the estimation of the risk to care homes in the presence of background community risk. This approach will be useful in facilitating the identification of the most vulnerable care homes and in predicting risk to new care homes.

The aim of this study was to demonstrate how two latent processes, community risk and care home risk can be modelled. The influence of community risk on care homes, offices, schools and gyms can be quantified using this method providing that the data is available.

Future work will focus on incorporating spatiotemporal data into the modeling approach. In particular, the combined effect of safety measures such as lockdown, vaccination of staff, and community risk will be incorporated in future work. Additionally, the incorporation of the level of uncertainty related to the use of relative risks from the Besag York Mollie model will be considered in extensions of the proposed modeling approach.

## Supporting information

S1 File (R)

S1 Data (ZIP)
