# Supplementary figures and images for: How does the community COVID-19 level of risk impact on that of a care home?
Source: PLoS One. 2021 Dec 31;16(12):e0260051. doi: 10.1371/journal.pone.0260051 (PMC8719703; doi:10.1371/journal.pone.0260051)

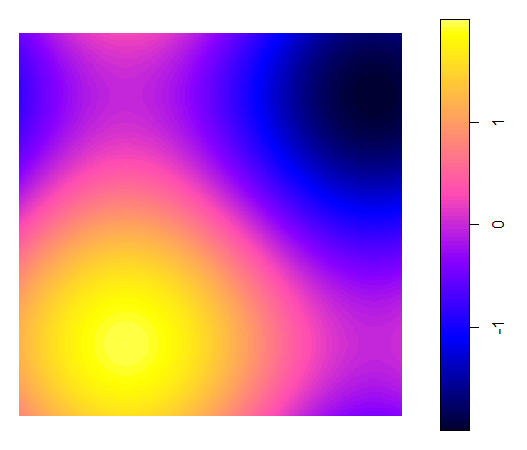

Supplement: S1 Data — (ZIP) [file pone.0260051.s002.zip › covariate.png]
